# The role of brain‐derived neurotrophic factor in learned fear processing: an awake rat fMRI study

**DOI:** 10.1111/gbb.12277

**Published:** 2016-01-05

**Authors:** A. P. Harris, R. J. Lennen, N. M. Brydges, M. A. Jansen, C. R. Pernet, H. C. Whalley, I. Marshall, S. Baker, A. M. Basso, M. Day, M. C. Holmes, J. Hall

**Affiliations:** ^1^ University/BHF Centre for Cardiovascular Sciences; ^2^ Centre for Cognitive Ageing and Cognitive EpidemiologyUniversity of EdinburghEdinburghUK; ^3^ Neuroscience and Mental Health Research InstituteCardiff UniversityCardiffUK; ^4^ Centre for Clinical Brain Sciences (CCBS) Neuroimaging Sciences; ^5^ Division of Psychiatry, Royal Edinburgh HospitalUniversity of EdinburghEdinbughUK; ^6^ AbbVie, Translational Sciences‐ImagingNorth ChicagoILUSA; ^7^ Alexion PharmaceuticalsCheshireCTUSA

**Keywords:** Amygdala, awake rat fMRI, BDNF, Pavlovian conditioning, transgenic rat

## Abstract

Brain‐derived neurotrophic factor (BDNF) signaling is implicated in the etiology of many psychiatric disorders associated with altered emotional processing. Altered peripheral (plasma) BDNF levels have been proposed as a biomarker for neuropsychiatric disease risk in humans. However, the relationship between peripheral and central BDNF levels and emotional brain activation is unknown. We used heterozygous BDNF knockdown rats (BDNF ^+/−^) to examine the effects of genetic variation in the BDNF gene on peripheral and central BDNF levels and emotional brain activation as assessed by awake functional magnetic resonance imaging (fMRI). BDNF ^+/−^ and control rats were trained to associate a flashing light (conditioned stimulus; CS) with foot‐shock, and brain activation in response to the CS was measured 24 h later in awake rats using fMRI. Central and peripheral BDNF levels were decreased in BDNF ^+/−^ rats compared with control rats. Activation of fear circuitry (amygdala, periaqueductal gray, granular insular) was seen in control animals; however, activation of this circuitry was absent in BDNF ^+/−^ animals. Behavioral experiments confirmed impaired conditioned fear responses in BDNF ^+/−^ rats, despite intact innate fear responses. These data confirm a positive correlation [r = 0.86, 95% confidence interval (0.55, 0.96); P = 0.0004] between peripheral and central BDNF levels and indicate a functional relationship between BDNF levels and emotional brain activation as assessed by fMRI. The results demonstrate the use of rodent fMRI as a sensitive tool for measuring brain function in preclinical translational studies using genetically modified rats and support the use of peripheral BDNF as a biomarker of central affective processing.

Brain‐derived neurotrophic factor (BDNF) is a widely expressed neurotrophin that signals through the tropomyosin‐related kinase B receptor (TrkB) to exert effects on neurogenesis, neuroprotection and synaptic plasticity (Poo 2001). In humans, reduced BDNF function is implicated in the pathophysiology of a range of neurological, affective and psychiatric disorders, including major depressive disorder, post‐traumatic stress disorder (PTSD) and schizophrenia (e.g. Schumacher *et al.* 2005; Weickert *et al.* 2003). In particular, altered levels of peripheral (serum) BDNF have been found in patients with depression, generalized anxiety disorder and PTSD, supporting BDNF's potential role as a biomarker for disease (Fernandes *et al.* 2014; Matsuoka *et al.* 2013; Wang *et al.* 2015). However, the relationship between peripheral BDNF levels, central BDNF levels and emotional brain function is currently unclear and is highly challenging to study directly in humans.

There is evidence that rats may be a particularly appropriate species with which to model key aspects of BDNF physiology of relevance to human disorders. Firstly, BDNF is present in the serum of humans and rats, but not of mice (Klein *et al.* 2011). This is an important difference given that serum BDNF levels may prove to be useful biomarkers of risk for psychiatric diseases in humans. And secondly, the larger brain size of rats makes them more tractable for certain physiological studies including brain imaging (Brydges *et al.* 2013).

Pavlovian fear conditioning is a robust method for studying emotional learning in rodents. Pavlovian fear associations are acquired by pairing a neutral stimulus (conditioned stimulus; CS e.g. a tone/visual cue or context) with an intrinsically aversive stimulus (unconditioned stimulus, US, e.g. foot‐shock) resulting in conditioned responding to subsequent presentations of the CS. Previous fear‐conditioning studies in rats and transgenic mice have revealed a key role for central BDNF in fear conditioning (Endres & Lessmann 2012; Hall *et al.* 2000; Liu *et al.* 2004; Rattiner *et al.* 2004; Soliman *et al.* 2010). Given the relevance of BDNF to human emotional disorders and the translational potential of transgenic rats, in this study, we investigated emotional learning in a genetically modified rat model of reduced BDNF expression.

Functional magnetic resonance imaging (fMRI), which uses changes in cerebral blood flow and oxygenation to provide a proxy for neuronal activation, is a potentially powerful translatable marker of brain function. fMRI has been used to investigate the neural underpinnings of affective disorders that associate with BDNF polymorphisms in humans (Egan *et al.* 2003; Montag *et al.* 2008; Soliman *et al.* 2010). In particular, fMRI has revealed that humans with BDNF polymorphisms have altered fear processing during fear‐conditioning paradigms (Soliman *et al.* 2010). We have developed fMRI methods for investigating brain responses to fear‐conditioned stimuli in awake rodents (Brydges *et al.* 2013; Harris *et al.* 2015). Here, we apply this approach to investigate the effect on brain activation of genetic knockdown of BDNF in transgenic rats.

Based on previous studies we predicted: (1) decreased peripheral and central BDNF levels in BDNF^+/−^ rats compared with littermate control rats, (2) decreased emotional learning in BDNF^+/−^ rats and (3) decreased activation of brain regions involved in emotional learning in response to conditioned stimuli presented during awake fMRI in BDNF^+/−^ rats, in particular the amygdala and insular regions (Brydges *et al.* 2013; Harris *et al.* 2015). Overall, this study aimed to provide a clearer understanding of the impact of genetic variation in BDNF on peripheral and central BDNF levels and emotional brain function.

## Methods and materials

### Animals: genotyping and husbandry

Animals were bred in‐house by crossing male rats heterozygous for a BDNF knockdown mutation (HET, SD‐BDNF^tm1sage^) generated using zinc finger nuclease technology (SAGE®Labs, St Louis, MO, USA) on a Sprague–Dawley (Hsd:SD) background with control female Sprague–Dawley rats (SAGE®Labs, St Louis, MO, USA). Resultant litters comprised of BDNF^+/+^ rats (controls) and heterozygous BDNF knockdown rats (BDNF^+/−)^ in a 1:1 ratio. Genotype was determined from a small ear biopsy taken at weaning following the protocol of SAGE®Labs. The experimenter was blind to genotype throughout the experiment.

BDNF^+/−^ and control (male rats only), weighing 300 ± 20 g (approximately 10 weeks old) at the start of the experiment, were housed in small groups (3–4 mixed genotypes per cage) with water and standard chow available *ad libitum*, in a humidity (50–60%) temperature (21°C) and light (on 0700–1900 h) controlled environment. All testing took place during the light phase. Prior to scanning, all rats were handled daily for 15 ± 5 days (15 min/day in groups of 4–5 at a time) to minimize stress associated with handling. Age at scanning was at least 12 weeks. All animal experiments were approved by the University of Edinburgh Ethical Review Committee, and studies were carried out in strict accordance with the UK Home Office Animals (Scientific Procedures) Act 1986 and the European Communities Council Directive of 22 September 2010 (Directive 2010/63/EU).

### Measurement of BDNF and TrkB

#### Group 1 rats

BDNF protein levels were measured in serum and whole hippocampus from experimentally naïve control and BDNF^+/−^ rats (n = 6/genotype; approximately 15 weeks old). Serum was processed from trunk blood, and whole hippocampus was dissected from the brain of the same animal (pooled across left and right hemisphere) following decapitation between 0900 and 1200 h. Samples were stored at −80°C until processing. BDNF protein was measured using a commercially available ELISA kit following the manufacturer's protocol (BDNF Emax ImmunoAssay System, Promega Southampton, UK, Cat No.G7611). See Appendix S1 (Supplementary Information) for sample preparation details. Serum BDNF levels were calculated as nanogram per milliliter (ng/ml) and brain levels as nanogram per gram (ng/g) per wet weight of tissue (ng/g ww). The linear correlation between serum and hippocampal BDNF protein levels was tested using Pearson's *r* following tests for normality, outliers and equal variance.

#### Group 2 rats

BDNF protein was measured in amygdala tissue punches (pooled across left and right hemisphere) from control and BDNF^+/−^ rats that had been fMRI scanned (n = 8 /genotype; culled approximately 2 weeks after fMRI) using the same ELISA kit as above. Levels are reported as ng/g per wet weight of tissue (ng/g ww).

#### Group 3 rats

Hippocampal BDNF and TrkB receptor mRNA levels were measured in a separate cohort of experimentally naïve rats (*n* = 6 control and 8 BDNF^+/−^ 15 weeks old). Hippocampal RNA was extracted using an RNeasy mini kit (Qiagen, Manchester, UK, cat No. 74104) following the manufacturers protocol and real time PCR was carried out on cDNA using a LightCycler 480 (Roche Diagnostics, Burgess Hill, UK) with a commercial master mix (FAM‐hydrolysis probe, Roche Diagnostics). See Appendix S1 for primer and probe details. Data from all three groups were normally distributed, had equal variance, no outliers and were analyzed using unpaired two‐sided *t*‐tests.

### Fear‐conditioning protocol

We initially confirmed that foot‐shock sensitivity was matched between genotype groups (See Fig. S1). Then behavioral assessment of fear conditioning was assessed in a cohort of animals not used for fMRI (*n* = 11 control; *n* = 17 BDNF^+/−^ rats). Rats were placed in the conditioning chamber (30 × 25 × 32 cm, Coulbourn Instruments, Whitehall, PA, USA) and over the course of a 25‐min period were exposed to five pairings (every 5 ± 1 min) of the CS: a 10 second flashing blue light (5 Hz max intensity flashes, 50/50 duty cycle) that co‐terminated with the US: a 0.5 second, 0.8 mA foot‐shock delivered through the grid floor. Percentage of time spent freezing during the inter shock intervals (ISI) was measured manually, directly and continuously and analyzed by repeated measures analysis of variance (RM ANOVA).

The conditioned response was assessed by measuring percentage of time freezing in response to the CS 24 h later in a novel context. The CS was presented (flashing light identical to training) for 2 min followed by 2 min rest (i.e. no light), repeated a total of 3 times. Freezing was measured manually, directly and continuously throughout each 2 min CS and rest period. The percentage time freezing during CS and rest periods was normally distributed and had equal variances and was analyzed by RM ANOVA.

### fMRI: acclimatization to the MRI scanning environment

**Figure 1:**
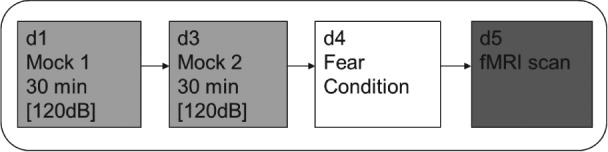
**Experimental timeline.** Rats were ‘mock scanned’ on days 1 and 3, fear conditioned on day 4 and retrieval of the conditioned fear response took place 24 h later during fMRI on day 5. The sound level (in dB) of the pulse sequence is given.

Prior to fMRI scanning, rats were acclimatized to the head and body restraint [Animal Imaging Research MRI (AIRMRI) Westminster, CA, USA; formerly Insight MRI; see Appendix S1 for details of the restraint apparatus) and MRI pulse sequence noise (120 dB) in mock scans that took place in a custom built mock scanner and lasted 30 min on days 1 and 3 (Brydges *et al.* 2013; King *et al.* 2005). Conditioning was conducted as described above on day 4. fMRI scanning took place in the MRI scanner on day 5 (See Fig. 1 for experimental timeline). For mock and MRI scanning, rats were lightly anesthetized (2–3% isoflurane in air and oxygen; 50:50 at 1 l/min) while placed in and out of the restraint apparatus. The rat in the restraint apparatus was then placed in the bore of the mock or MRI scanner as appropriate.

### fMRI: image acquisition

In total, 13 BDNF^+/−^ and 14 control rats were scanned (sample size based on previous rodent fMRI studies which revealed significant fear circuitry activation (Brydges *et al.* 2013; Harris *et al.* 2015). Twenty‐four hours after cued fear conditioning, rats were placed into the restraint apparatus. An adjustable surface coil was fixed inside the head restraint and pressed firmly on the rat's head and locked in place. The rat was then placed into a 7 T MRI scanner (Agilent Technologies, Oxford, UK) equipped with a 400 mT/m gradient set, a 72 mm volume RF coil for transmission and a surface coil for signal reception (AIRMRI). Structural images were acquired under 2–3% isoflurane using fast spin echo (FSE) sequence with a matrix of 256 × 256, field of view 30 mm (in plane resolution 117 µm), repetition time = 3000 ms, echo train length 8, effective echo time = 48 ms, 4 signal averages and 26 × 1 mm coronal slices and took 12 min to acquire. Animals were then allowed to recover from anesthesia (breathing rate > 100/min, which typically took approximately 10 min) before beginning the functional scanning paradigm. Isoflurane is rapidly eliminated from the body (Ferris *et al.* 2006) and the first CS exposure occurred 15–17 min following isoflurane withdrawal. If isoflurane effects were still present in the brain following withdrawal (e.g. Thrane *et al.* 2012), these effects would be similar in the BDNF^+/−^ and control rats. Once awake, functional scanning began and after a 5 min acclimatization period the CS was presented in the scanner using a custom built array of high intensity blue LEDs. The test paradigm consisted of 2 min flashing light (CS) alternating with 2 min ‘rest’ period (during which the CS was not presented) this was repeated a total of three times (without any shocks being administered) with randomized starting order. Functional image acquisition used an FSE sequence with a matrix of 64 × 64 (in plane resolution 469 µm), repetition time = 2500 ms, echo train length 16 and effective echo time = 36 ms. Each functional volume consisted of 16 × 1 mm slices covering the brain (excluding the cerebellum) and took 10 seconds to acquire. Functional scanning was run for a total of 103 volumes and took 17 min and 10 seconds to complete.

### fMRI: data preprocessing and analysis

SPM8 (Wellcome Trust Centre for NeuroImaging, University College London, UK) was used for image analysis. To facilitate analysis in SPM8, all images were scaled up by a factor of 10 in the x, y and z dimensions to account for the relative size difference between human and rat brain. For each rat, functional images were realigned to remove minor motion, and a mean functional volume generated. (At this stage, two control rats were removed from the analysis due to translational movement >1.5 mm and rotational movement >5 mrads; displacement was calculated as detailed (Van Dijk *et al.* 2012). Levels of displacement did not differ between genotypes; see Table S1). The rat's structural image was then co‐registered to the corresponding mean functional image. Segmentation of the structural image, using tissue probability maps for gray and white matter and cerebrospinal fluid (Valdes‐Hernandez *et al.* 2011) was then combined with spatial normalization to the template using a resampled voxel size of 2 × 2 × 2 mm to match the template (Valdes‐Hernandez *et al.* 2011), and the same normalization parameters were applied to the functional images. Finally, all realigned and normalized images were smoothed with a 6 × 6 × 6 mm full width at half maximum Gaussian filter to increase signal to noise and allow for small anatomical and functional variations between rats (Mikl *et al.* 2008).

First level analysis was performed on all rats using a general linear model in SPM8. The three CS presentations were modeled as a single regressor and the 5 min acclimatization period and rest periods were modeled as an implicit baseline in the model. Parametric modulation was used to model a linear decrease from the first CS to the third CS to investigate any increase or decrease in activation. All data were modeled by a boxcar convolved with the SPM8 canonical haemodynamic response function. Movement parameters generated in the re‐alignment step were added in to the model as multiple regressors and event‐dependent high‐pass filtering was used whereby the cutoff period was 580 seconds. Temporal autocorrelation was accounted for using the AR(1) whitening model of SPM8. Parameter images corresponding to the CS > baseline regressor were entered into a second level random effects model, in which within group effects (e.g. CS > baseline; CS1 > CS3) were examined using one‐sample *t*‐test and between group effects (e.g. control > BDNF^+/−^) were examined using a two sample *t*‐test. Whole brain group level statistical parametric maps (SPMs) had cluster forming thresholds of *P* < 0.001, and clusters were considered significant at *P* < 0.05 corrected for multiple comparisons using cluster level correction based on random field theory (*P*_FWE_: family wise error corrected P value). SPMs are presented overlaid onto an average structural image (created using the average of all normalized structural images from this study). The cluster level *P*_FWE_; number of voxels within the cluster; peak T value and co‐ordinates in x, y and z dimensions of the peak voxel within the cluster are reported. The co‐ordinates (millimeters) refer to the Bregma coordinate system (the origin point is where Bregma zero intersects with the center of the anterior commissure, n.b. data are scaled by 10; x = left/right, y = anterior/posterior, z = ventral/dorsal).

Unthresholded activation maps were generated to investigate visual activation in response to the CS. These maps show the raw effect size for each voxel and thus show all regions (uncorrected for multiple comparisons) for which there was some activation (Jernigan *et al.* 2003), but because these clusters do not pass the strict FWE cluster correction used here, they cannot be reported as significant.

### Correlation between amygdala BDNF level and amygdala function

To investigate the relationship between amygdala activation and amygdala BDNF levels, parameter estimates (first eigenvalues) were extracted from the functional cluster over the left amygdala (using contrast CS > baseline for control rats) and then correlated with the corresponding amygdala BDNF level. The parameter estimates represent an estimate of the relative response amplitude of neural activations elicited during the different conditions (e.g. CS > baseline; baseline = 0).

### Laterality and habituation of amygdala activation to the CS

To explicitly investigate laterality and habituation of amygdala activation across the three CS presentations, a second GLM was used to model the three CS as separate regressors and the rest periods as an implicit baseline. Anatomically defined masks (covering the whole left and right amygdala complex) were created using MRIcro (Rorden & Brett 2000) using the Paxinos and Watson Atlas as a guide (Paxinos & Watson 2004). The parameter estimates (first eigenvalues) were then extracted per region of interest. The parameter estimates from the first, second and third CS presentations for the control and BDNF^+/−^ rats were then analyzed by RM ANOVA, genotype was a main effect and CS presentation (CS1, 2, 3) and hemisphere (left/right) were repeated measures.

### Elevated plus maze

Innate anxiety was measured in a separate cohort of rats (*n* = 10 control; *n* = 11 BDNF^+/−^ rats; 16 weeks old) in an elevated plus maze between 0900 and 1500 h. Rats were placed individually on the maze and left to explore for 5 min (maze and protocol details; Brydges *et al.* 2012). Behavior was recorded using ANY‐maze tracking software (ANY‐maze, Stoelting Co., Wood Dale, IL, USA). The percentage time in the open arms, percentage distance moved in the open arms and number of crossings into the open arms over the 5 min trial was compared between the genotypes by unpaired two‐sided *t*‐test. A blood sample was taken, in a separate room, by tail nick and collected in a tube pretreated with  ethylenediaminetetraacetic acid  immediately following EPM testing for plasma corticosterone measurement using an in‐house radioimmuno‐assay using [3H]‐corticosterone (assay details; Aldujaili *et al.* 1981). All data were normally distributed and had equal variance and were analyzed using unpaired two‐sided *t*‐test.

## Results

### BDNF and TrkB levels

Serum BDNF protein levels were 73% lower in BDNF^+/−^ rats compared with control rats [−0.87 ng/ml; 95% CI (−1.2, −0.59); *t*_10_ = 6.8, *P* < 0.0001; Table 1]. Central BDNF protein levels were also significantly lower in BDNF^+/−^ compared with control rats; 31% lower in the hippocampus [−1.1 ng/g ww; 95% CI (−1.5, −0.7); *t*_10_ = 5.6, *P* = 0.0002; Table 1], and 45% lower in the amygdala [−1.3 ng/g ww; 95% CI (−2.3, −0.3); *t*_11_ = 2.8, *P* = 0.017, Table 1]. There was a significant positive correlation between serum and hippocampus BDNF levels [Pearson's correlation coefficient: *r* = 0.86, 95% CI (0.55, 0.96); *P* = 0.0004; see Fig. S2a]. The mRNA levels of BDNF and TrkB in the hippocampus did not differ between the genotypes (*P* > 0.05; Table 1).

**Table 1:** BDNF protein in serum, hippocampus and amygdala homogenates and BDNF and TrkB receptor mRNA levels in hippocampal homogenates (± SEM)

Parameter	Control rats	BDNF^+/−^ rats
Serum BDNF protein (ng/ml)	1.1 ± 0.1	0.3 ± 0.04***
Hippocampal BDNF protein (ng/g ww)	3.6 ± 0.1	2.5 ± 0.2***
Amygdala BDNF protein (ng/g ww)	2.9 ± 0.3	1.6 ± 0.4*
Hippocampal BDNF mRNA (AU)	0.9 ± 0.1	0.9 ± 0.1
Hippocampal TrkB mRNA (AU)	1.1 ± 0.1	1.3 ± 0.1

AU, arbitrary units.

* *P* < 0.05 unpaired *t*‐test;

*** *P* < 0.001.

### BDNF ^+/−^ rats have an impaired freezing response to a cued CS

**Figure 2:**
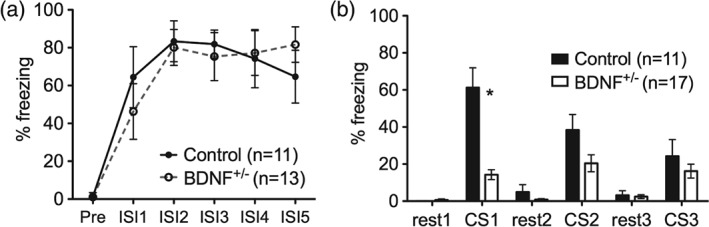
**Behavioral assessment of cued fear conditioning.** (a) Percentage time freezing (± SEM) during acquisition of cued fear conditioning before the first shock (pre) and during the ISI (1–5). (b) Percentage time freezing (± SEM) during retrieval, 24 h after conditioning. The controls (black bars; n = 11) spent significantly longer freezing in response to the first presentation of the CS than the BDNF^+/−^ rats (white bars; n = 17; * Tukey HSD post hoc P < 0.05).

During acquisition of the cued fear‐conditioning freezing  during the ISI significantly increased in both control  littermate and BDNF^+/−^ rats (main effect of ISI: F_5,110_ = 78.9, *P* < 0.0001; Fig. 2a). During retrieval, 24 h after cued fear conditioning, all rats showed increased freezing behavior in response to CS presentation confirming that control and BDNF^+/−^ rats detected the visual stimulus, however, the response to the first CS was significantly reduced in the BDNF^+/−^ rats compared with controls (genotype‐by‐CS interaction: F_2,48_ = 12.5, *P* < 0.0001; Tukey HSD *post hoc* for CS1: *P* < 0.05; Fig. 2b). The control rats showed a significant decrease in freezing over the 3 CS presentations (CS presentation: F_2,20_ = 7.9, *P* = 0.003; Fig. 2b).

### Control rats activate fear circuitry in response to a cued CS

**Figure 3:**
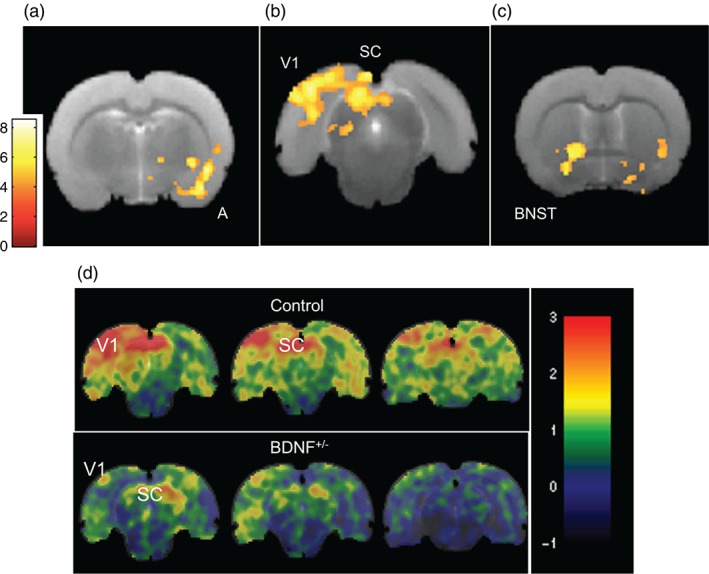
**SPMs of brain activation (BOLD signal) in response to the CS versus baseline in control rats (n = 12) and unthresholded activation maps for control and BDNF^+/−^ rats.** (a) A significant cluster was found over the left anterior amygdala [A; P_FWE_ < 0.0001, K_E_ = 853, peak T = 8.55 at (38, −16, −2)], (b) the right primary visual cortex and superior colliculus [V1, SC; P_FWE_ < 0.0001, K_E_ = 1568, peak T = 7.45 at (−48, −72, 44)] and (c) bed nucleus of the stria terminalis reaching forward to the right nucleus accumbens [BNST; P_FWE_ = 0.002, K_E_ = 327, peak T = 6.71 at (−24, 2, −2)]. Cluster forming threshold: P < 0.001, maps presented overlaid on the average structural image (n = 25), the right side of the image is the left side of the brain. Scale bar represents T‐score. (d) Unthresholded activation maps for control (n = 12) and BDNF^+/−^ rats (n = 13) show activation in the primary visual cortex (V1) and SC in response to the CS. Color bar represents the raw effect size (increase/decrease) in arbitrary units.

In the fMRI experiment, within group analysis of the control rats revealed one large cluster of activation which extended over the left amygdala (encompassing the basolateral, medial and lateral nuclei), into the hypothalamus region and up into the left granular insular and somatosensory cortex, key regions implicated in processing fear [*P*_FWE_ <0.0001, K_E_ = 853, peak T = 8.55 at (38, −16, −2); Fig. 3a; Table 2]. The primary visual cortex, superior colliculus, bed nucleus of the stria terminalis, restrosplenial dysgranular cortex and caudate putamen were also activated during the CS presentations (Fig. 3b and 3c; Table 2). No significant clusters of activation were found when modeling a linear decrease in brain activation from CS1 to CS3 using parametric modulation. The within group analysis of the BDNF^+/−^ rats in response to the CS revealed no significant fear circuitry and no significant clusters of activation were found when modeling a linear decrease in brain activation from CS1 to CS3 using parametric modulation.

**Table 2:** Clusters of brain activation (BOLD response) in the control (n = 12) and BDNF^+/−^ rats (n = 13)

Cluster statistic P_FWE corrected_	Cluster extent	Peak T score in cluster	Co‐ordinates of peak voxel (mm)	Description of region
Control within group (CS > baseline), cluster forming threshold *P* < 0.001				
*P* < 0.0001	853	8.55	26, −20, −18	L amygdala, SSC, GI
*P* = 0.003	301	8.00	38, −16, −2	L ant. caudate putamen
*P* < 0.0001	1568	7.45	−48, −72, 44	R primary visual ctx, SC
*P* < 0.0001	452	7.21	−6, −54, 42	RSD ctx
*P* = 0.002	327	6.71	−24, 2, −2	R BNST
Control > BDNF^+/−^ between groups (CS > baseline), cluster forming threshold *P* < 0.001				
*P* = 0.001	490	6.49	46, −12, −20	L amygdala
*P* = 0.06	195	5.38	58, 12, −2	L GI
*P* = 0.057	198	4.91	−48, −76, 30	R parasubiculum
*P* = 0.062	193	4.79	6, −52, 38	RSD ctx and PAG

ant, anterior; BNST, bed nucleus of the stria terminalis; ctx: cortex; GI, granular insular cortex; L, left; PAG, periaqueductal gray; R, right; RSD, retrosplenial dysgranular cortex; RSD ctx, retrosplenial dysgranular cortex; RSGc: retrosplenial granular cortex; SC, superior colliculus; SSC, secondary somatosensory cortex; T, peak voxel *t*‐statistic.

Coordinates in millimeters refer to the Bregma coordinate system (anterior commissure, n.b. data were scaled by 10).

Unthresholded activation maps revealed that the superior colliculus and the primary visual cortex were activated in response to the CS in the control and BDNF^+/−^ rats (Fig. 3d). Furthermore, parameter estimates extracted from the primary visual cortex and superior colliculus did not significantly differ between control and BDNF^+/−^ (Table S2) supporting activation of visual processing regions in both groups, but since the clusters in the BDNF^+/−^ rats did not pass the strict FWE cluster correction used here, they cannot be reported as significant.

### Control rats have greater fear circuitry activation in response to a cued CS than BDNF ^+/−^ rats

**Figure 4:**
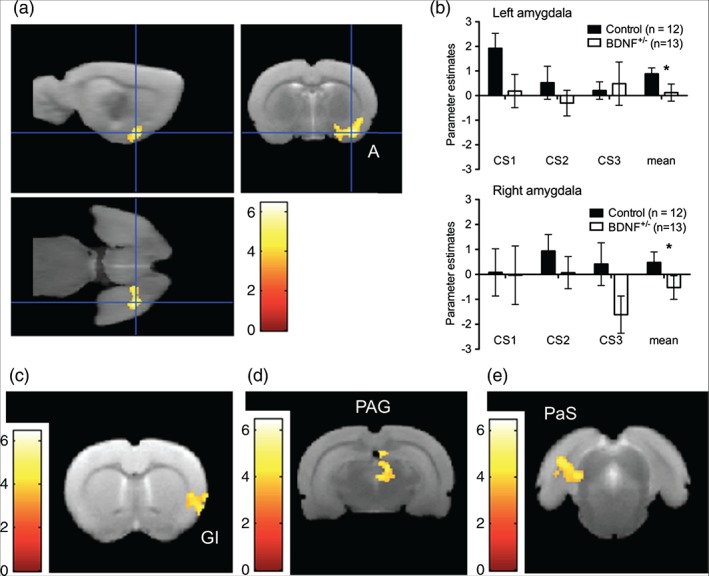
**SPMs showing brain activation (BOLD signal) in response to the CS versus baseline that was greater in control rats (n = 12) than in BDNF^+/−^ rats (n = 13).** (a) Coronal, sagittal and axial views of the cluster over the left amygdala [A; P_FWE_ < 0.001, K_E_ = 490, peak T = 6.49 at (46, −12, −20)]. (b) Parameter estimates from left and right amygdala across the three CS presentations; *main effect of genotype P < 0.05. (c) The GI [P_FWE_ < 0.06, K_E_ = 195, peak T = 5.38 at (58, 12, −2)], (c) the PAG [P_FWE_ = 0.062, K_E_ = 193, peak T = 4.79 at (6, −52, 38)] and e) the right parasubiculum [PaS; P_FWE_ = 0.057, K_E_ = 198, peak T = 4.91 at (−48, −76, 30)]. SPMs are presented overlaid on the average structural image (n = 25) and the right side of the image is the left side of the brain. Scale bar represents T‐score.

We next directly compared brain activation in response to the CS between control and BDNF^+/−^ rats. Between‐group analysis revealed a highly significant cluster covering the left amygdala complex that showed greater activation in control than the BDNF^+/−^ rats [*P*_FWE_ < 0.0001, K_E_ = 490, peak T = 6.49 at (42, −10, −22); Fig. 4a; Table 2]. Parameter estimates were also significantly higher in the control rats compared to BDNF^+/−^ rats to the CS, irrespective of hemisphere (main effect of genotype: F_1,23_ = 5.8, *P* = 0.025; Fig. 4b).

The between‐group analysis revealed three other clusters that showed greater activation in control than the BDNF^+/−^ rats in regions implicated in fear processing, however, these clusters only reached a trend for statistical significance when corrected for multiple comparisons across the whole brain (cluster level *P*_FWE_ < 0.06). One cluster was found in the left granular insular (GI), which comprises a component of the pain pathway recruited into processing conditioned fear [*P*_FWE_ = 0.06, K_E_ = 195, peak T = 5.38 at (58, 12, −2); Fig. 4c]. The second cluster was found in the dorsomedial periaqueductal gray region (PAG; reaching up into the superior colliculus) which is involved in the processing of defensive behavior (e.g. freezing) in response to the CS [PaS; *P*_FWE_ = 0.062, K_E_ = 193, peak T = 4.79 at (42, −10, −22); Fig. 4d]. The third cluster was found in the right parasubiculum [*P*_FWE_ = 0.057, K_E_ = 198, peak T = 4.91 at (−48, −76, 30); Fig. 4e]. These data support greater fear circuitry activation in the controls than in the BDNF^+/−^ rats in response to the CS.

Examination of neural activation that was greater in the BDNF^+/−^ animals than in control rats in response to the CS did not reveal any significant clusters when corrected for multiple comparisons across the whole brain. There were no significant differences between the genotypes when investigating activation that was greater in CS1 than in CS3 (i.e. modeling extinction).

### Amygdala activation correlates with amygdala BDNF levels

There was a significant positive correlation between the extracted parameter estimates from the functional left amygdala cluster and baseline amygdala BDNF levels [measured 2 weeks after fMRI; Pearson's correlation coefficient r = 0.73, 95% CI for slope (0.3, 0.9); *P* = 0.0044; See Fig. S2b]. This supports the hypothesis that animals with low basal levels of BDNF in the amygdala have reduced amygdala activation to a cued CS.

### Laterality and habituation of amygdala activation to the CS

Parameter estimates extracted from the whole amygdala region in the left and right hemisphere were not significantly different, supporting bilateral amygdala activation to the CS (effect of hemisphere: F_1,23_ = 1.7, *P* = 0.20; Fig. 4b). All interactions with CS (1–3) and hemisphere were not significant.

Based on the result of the behavioral experiment (in which control rats displayed reduced freezing in CS3 compared to CS1; Fig. 2b), parameter estimates from the left amygdala in response to CS1 were compared with CS3 using paired *t*‐test. There was a trend for decreased left amygdala activation in control rats (paired *t*‐test: *t*_11_ = 2.1, *P* = 0.057), supporting a tendency for amygdala habituation across the CS presentations.

### Spontaneous (unconditioned) anxiety is unchanged in BDNF ^+/−^ rats

There were no significant differences between BDNF^+/−^ and control rats in any of the parameters measured on the elevated plus maze (Table S3). Levels of the stress hormone corticosterone, measured immediately following testing, did not significantly differ between the genotypes (*t*_18_ = 0.9, *P* = 0.37; Table S3). These data suggest equal levels of spontaneous innate anxiety‐like behavior and stress hormone responses in response to stress in BDNF^+/−^ and control rats.

## Discussion

In this study, we show reduced central and peripheral BDNF levels and impaired emotional brain activation during awake fMRI in BDNF^+/−^ rats compared with controls. BDNF protein levels were significantly reduced in the serum, hippocampus and amygdala of BDNF^+/−^ rats relative to controls, with no accompanying compensatory changes in the BDNF receptor, TrkB (Gururajana *et al.* 2014; St. Laurent *et al.* 2013). In addition, central BDNF levels strongly correlated with peripheral BDNF levels. Using awake rat fMRI, we were able to demonstrate decreased amygdala activation to an emotional conditioned cue in BDNF^+/−^ rats compared with controls, reflecting the decrease in amygdala BDNF levels.

It is widely accepted that the amygdala is a critical structure for the acquisition, storage and expression of learned fear (LeDoux 2000). Presentation of a cue previously associated with foot‐shock in our fMRI experiment resulted in the activation in control animals of a cluster that encompassed the amygdala complex [basolateral amygdala (BLA) and central nucleus (CeA) of the amygdala in the left hemisphere]. The BLA receives sensory information and plays a key role in forming the CS–US association (reviewed in Maren 2001). The CeA is then held to mediate the output from the amygdala to downstream structures to coordinate the behavioral and physiological response to the CS (e.g. freezing, increased heart‐rate). In addition, parameter extracts from the left amygdala showed a tendency to decrease in response to CS3 compared with CS1, supporting habituation of fear circuitry activation over time, as found previously in both rodent and human fear‐conditioning fMRI studies (Brydges *et al.* 2013; Buchel *et al.* 1998).

Although lateralisation of amygdala activation in response to emotional processing appears to be consistently found in both rodent and human fMRI studies (Brydges *et al.* 2013; Harris *et al.* 2015; LaBar *et al.* 1998; Pine *et al.* 2001) reports of laterality must be supported by an explicit test of the hemisphere‐by‐task interaction. In this experiment, the activation maps suggested unilateral amygdala activation in response to the CS, however, a formal test of the hemisphere‐by‐task interaction on the extracted parameter estimates from the whole amygdala region did not support this, as found previously in our laboratory in mice (Harris *et al.* 2015). Brydges *et al.* (2013) reported activation in the right amygdala but did not formally test for laterality, therefore, both sides may have been activated but below the statistical threshold (as in this experiment).

In addition to the amygdala, we also found evidence of activation in response to the CS in control animals of the somatosensory cortex and granular insular cortex, both of which can project to the BLA complex and form a component of a pain processing pathway that is recruited into the fear network in response to the CS (reviewed in McDonald 1998). We additionally observed activation in control animals of the periaquaductal gray region, which receives direct projections from the CeA and is reported to play a role in processing defensive behavior (e.g. freezing) (Behbehani 1995; Rizvi *et al.* 1991) and the primary visual cortex and superior colliculus, which likely reflects visual processing of the stimulus (Van Camp *et al.* 2006). Activation was also seen in controls in the bed nucleus of the stria terminalis, which is a major output pathway of the amygdala.

BDNF^+/−^ rats showed a decreased behavioral response (freezing) and decreased activation of the amygdala and related fear processing circuitry in response to the CS in our fMRI experiment (Korte *et al.* 1995; Minichiello *et al.* 1999). These results demonstrate impaired amygdala‐dependent learning in these animals and show that in this model reduced peripheral and central BDNF is associated with impaired function of brain regions involved in processing conditioned emotional stimuli (Endres & Lessmann 2012; Korte *et al.* 1995; Minichiello *et al.* 1999). Foot‐shock sensitivity and the ability to detect visual cues (as tested in the behavioral fear‐conditioning experiment) were not impaired in the BDNF^+/−^ rats, eliminating these factors as potential confounds for the behavioral and imaging findings. Furthermore, unthresholded activation maps showed that visual processing regions (visual cortex and superior colliculus; Van Camp *et al.* 2006) were activated in response to the CS in BDNF^+/−^ and control rats (Fig. 3d). Although the visual activation in the BDNF^+/−^ rats was below the statistical threshold when corrected for multiple comparisons across the whole brain, it was not significantly different to that seen in control rats.

Innate (i.e. unconditioned) anxiety, as tested on the elevated plus maze, was not affected by reduced BDNF levels in our experiment. This finding is consistent with the results from previous studies using BDNF^+/−^ rats and mice (Endres & Lessmann 2012; Gururajana *et al.* 2014; MacQueen *et al.* 2001; Montkowski & Holsboer 1997) and the percentage time spent in open arms (10%) is comparable with previous rat studies (Gururajana *et al.* 2014). Stress hormone levels in response to the mild stress of elevated plus maze testing were also unaltered in BDNF^+/−^ rats. Similarly, corticosterone levels at resting and following 30 min restraint stress are reported to remain unaltered in BDNF^+/−^ mice relative to controls (Chourbaji *et al.* 2003), these data along with our findings suggest that reduced BDNF levels do not alter HPA‐axis reactivity or feedback. It is plausible that innate anxiety is not dependent on BDNF levels because it does not require explicit new learning (unlike fear conditioning). These data taken together with the fMRI data suggest that impaired emotional learning, rather than altered innate anxiety and HPA‐axis reactivity, may accompany reduced BDNF levels in human psychiatric disorders.

Emotional disorders such as depression, generalized anxiety disorder and PTSD in humans have been shown to be associated with altered peripheral BDNF levels (Fernandes *et al.* 2014; Matsuoka *et al.* 2013; Wang *et al.* 2015), and these peripheral levels are in turn postulated to reflect central BDNF signaling which is assumed to influence brain function. However, this hypothesis is challenging to test directly in humans. Using genetically modified rats coupled with fMRI, we were able to confirm that baseline levels of BDNF in the amygdala are a significant predictor of amygdala function during emotional processing. While we cannot directly confirm that serum levels correlated with amygdala function, we and others (Klein *et al.* 2011; Pillai *et al.* 2010) report a strong correlation between peripheral and central BDNF levels, and therefore, we postulate that peripheral BDNF levels may be a valid biomarker for BDNF action in the brain and brain function, at least in the context of genetic variation affecting the BDNF gene.

In summary, BDNF^+/−^ rats represent a model of impaired plasticity across a range of neuropsychiatric conditions. We show that BDNF^+/−^ rats have reduced peripheral and central BDNF levels that are associated with changes in brain activation during the processing of conditioned emotional stimuli. These results demonstrate, for the first time, the use of fMRI to detect altered fear processing in awake *genetically modified* rats that are completing a learned emotional task. The results also provide evidence for a relationship between peripheral BDNF levels and emotional brain function, which is of relevance to human biomarker research. We conclude that awake fMRI with genetically modified rats provides a promising approach to screen potential biomarkers and therapies for affective disorders in humans.

## Supporting information


**Appendix S1:** Supporting data.
**Table S1:** Mean head motion (± SEM) between volumes during fMRI.
**Table S2:** Parameter estimates (± SEM) from the primary visual cortex and superior colliculus region for control and BDNF^+/−^ rats.
**Table S3:** Behavioral parameters (± SEM) on the open arm during elevated plus maze testing (EPM) and plasma corticosterone (CORT) levels immediately following testing for control (n = 10) and BDNF^+/−^ rats (n = 11).
**Figure S1:** Foot‐shock sensitivity in control and BDNF^+/−^ rats. Behavioural score (± SEM) during shock titration. The response of the rat was scored as follows: 0 = no response; 1 = slight flinch; 2 = flinch/tail flick; 3 = fast walking around chamber; 4 = jumping up with all feet off the grid floor; 5 = jumping up with vocalization and fast walking. 0.8 mA was used in the main experiment.
**Figure S2:** BDNF correlations. (a) There was a significant positive correlation between serum and hippocampus BDNF levels [Pearson's correlation coefficient: r = 0.86, 95% CI (0.55, 0.96); P = 0.0004] and (b) there was a significant positive correlation between the extracted parameter estimates from the functional left amygdala cluster and baseline amygdala BDNF levels [measured 2 weeks after fMRI; Pearson's correlation coefficient r = 0.73, 95% CI for slope (0.3, 0.9); P = 0.0044].
