# Supplementary material for: The role of brain‐derived neurotrophic factor in learned fear processing: an awake rat fMRI study
Source: Genes Brain Behav. 2016 Jan 5;15(2):221–30. doi: 10.1111/gbb.12277 (PMC4819698; doi:10.1111/gbb.12277)
Supplement: Supplementary file 1 — Appendix S1: Supporting data. Table S1: Mean head motion (± SEM) between volumes during fMRI. Table S2: Parameter estimates (± SEM) from the primary visual cortex and superior colliculus region for control and BDNF+/− rats. Table S3: Behavioral parameters (± SEM) on the open arm during elevated plus maze testing (EPM) and plasma corticosterone (CORT) levels immediately following testing for control (n = 10) and BDNF+/− rats (n = 11). Figure S1: Foot‐shock sensitivity in control and BDNF+/− rats. Behavioural score (± SEM) during shock titration. The response of the rat was scored as follows: 0 = no response; 1 = slight flinch; 2 = flinch/tail flick; 3 = fast walking around chamber; 4 = jumping up with all feet off the grid floor; 5 = jumping up with vocalization and fast walking. 0.8 mA was used in the main experiment. Figure S2: BDNF correlations. (a) There was a significant positive correlation between serum and hippocampus BDNF levels [Pearson's correlation coefficient: r = 0.86, 95% CI (0.55, 0.96); P = 0.0004] and (b) there was a significant positive correlation between the extracted parameter estimates from the functional left amygdala cluster and baseline amygdala BDNF levels [measured 2 weeks after fMRI; Pearson's correlation coefficient r = 0.73, 95% CI for slope (0.3, 0.9); P = 0.0044]. [file GBB-15-221-s001.docx]

***SUPPLEMENTAL INFORMATION***

**METHODS**

*Preparation of serum and brain tissue for BDNF ELISA*

Serum was diluted 1:8 with kit buffer, and results were calculated as ng/ml. Hippocampal and amygdala samples were homogenized using a 1:10 weight by volume (w/v) and 1:15 w/v dilution, respectively, in lysis buffer (100mM Tris-HCl pH 7.2, 400mM NaCl, 4mM EDTA, 0.05% sodium azide, 0.5% gelatin, 0.2% Triton-X, 2% BSA [Sigma, A7030] and Sigma complete protease inhibitor cocktail [Sigma, S8820] (Elfving et al. 2010). Samples were then acid treated following manufacturers protocol and then diluted in kit buffer (final dilution of 1:100 for hippocampus and 1:90 for amygdala).

*Probes/primers used in measurement of BDNF and TrkB mRNA*

The following primers and probe sets were used: TrkB forward 5’-cgaggttggaacctaacagc-3’ and reverse 5’-ccttttctggtttgcaatgag-3’ primers with Universal Probe Library (UPL; Roche Diagnostics) probe 115, BDNF forward 5’-gtggaggctaagtggagctg-3’ and reverse primers 5’-caggatggccactcagaaat-3’ with UPL probe 53. Gene of interest mRNA levels were normalized against hypoxanthine- phosphoribosyl transferase (HPRT), which did not differ between the genotypes. HPRT primers forward 5’-tcctcctcagaccgcttt-3’ and reverse 5’-cctggttcatcatcgctaatc-3’ primers with UPL probe 95.

*Measurement of foot-shock sensitivity in control and BDNF^+/-^ rats*

We ascertained sensitivity to foot-shock in a separate cohort of rats (n = 6 control; n = 4 BDNF^+/-^). A rat was individually placed in the conditioning chamber (30 cm x 25 cm x 32 cm; Coulbourn Instruments, PA, USA) and exposed to a 0.5 s shock with increasing strength (0.1 mA to 0.8 mA; increasing in 0.1 mA increments) (Baran et al. 2009). One shock was delivered every 2 min. The response of the rat was scored [0 = no response; 1 = slight flinch; 2 = flinch/tail flick 3 = fast walking around chamber; 4 = jumping up with all feet off the grid floor; 5 = jumping up with vocalization and fast walking]. The observer was blind to genotype but was aware of the strength of shock. Data were analysed by repeated measures (RM) ANOVA.

*Restraint apparatus used during fMRI acclimatization and scanning*

For mock and MRI scanning, rats were lightly anaesthetized (2-3% isoflurane in air and oxygen; 50:50 at 1L/min) whilst placed in and out of the restraint apparatus (Animal Imaging Research MRI [AIRMRI] Westminster, USA; formerly Insight MRI). A plastic semi-circular headband with blunted ear supports that fit into the ear canal was positioned over the head. The head was then placed into a cylindrical head holder with front teeth secured over a bite-bar and the ear supports were gently secured into lateral sleeves with adjustable screws (Ferris et al. 2005). Respiration was monitored during scanning by means of a pressure transducer placed under the torso (MR compatible Small Animal Monitoring and Gating System; SA Instruments Inc.) and a rectal thermistor probe was inserted to monitor and maintain body temperature at 37 ± 0.5°C by means of a feedback controlled warm air system (SA Instruments Inc.).

*Brain movement during fMRI scanning.*

Translation and rotation of the brain between volumes was estimated from movement parameters generated during rigid body correction of head motion in the preprocessing realignment step (Van Dijk et al. 2012, Harris et al. 2015). Rats that moved more than two thirds of a voxel (functional voxel size: 0.469 x 0.469 x 1mm) were removed from the analysis. Mean translation (mm) and rotation (mrads) for the control and BDNF^+/-^ rats was analysed by unpaired *t*-test to compare overall movement between the genotypes.

*Extraction of parameter estimates from visual processing regions*

Anatomically defined masks (covering the superior colliculus and primary visual cortex) were created using MRIcro (Rorden and Brett 2000) using the Paxinos and Watson Atlas as a guide (Paxinos and Watson 2004), and parameter estimates were extracted.

**RESULTS**

*Foot-shock sensitivity*

Sensitivity to foot-shock was similar between the control and BDNF^+/-^ rats (genotype: F_1,8_ = 1.5, *P* = 0.25; Fig. S1) and there was an increase in the ‘behavioral score’ of the response to shock that was comparable for both genotypes as the shock intensity increased (effect of shock increase: F_7,56_ = 31.0, *P* < 0.0001; Fig. S1). 0.8mA was used in the main experiment.

*Brain movement during fMRI*

Small rotations and translations were seen during the functional scanning, however, these did not exceed two thirds of a voxel size (0.469 x 0.469 x 1 mm) in the rats included in the analysis (two control rats were removed from the analysis due to excessive movement). After discounting the animals with excessive movement, mean displacement (mm) and mean rotation (mrads) did not differ significantly between the genotypes (translation: t_23_ = 0.21, *P* = 0.83; rotation: t_23_ = 1.1, *P* = 0.3; Table S1).

*Visual activation in response to the CS (region of interest analyses)*

Parameter estimates extracted from the superior colliculus (SC) and primary visual cortex (V1) did not differ between control and BDNF^+/-^ rats (difference between genotypes SC: 0.99; 95% CI [-0.7, 2.3]; two sample t-test: t_22_ = 1.2, *P* = 0.23; V1: 1.1; 95% CI [-0.4, 2.6]; two sample t-test: t_22_ = 1.6, *P* = 0.87; Table S3), the parameter estimates pooled across the genotypes were significantly different to zero (SC mean parameter estimate: 1.3; 95% CI [0.5, 2.2]; one sample t-test: t_23_ = 3.0, *P* < 0.005; V1 mean parameter estimate: 1.4; 95% CI [0.6, 2.1]; one sample t-test: t_23_ = 3.7, *P* < 0.001), confirming similar levels of activation in the superior colliculus and primary visual cortex to the CS in BDNF^+/-^ and control rats.

**TABLES**

**Table S1. Mean head motion (**± **SEM) between volumes during fMRI.** Translation and rotation of the brain between volumes was estimated from movement parameters generated during rigid body correction of head motion in the preprocessing realignment step (Van Dijk et al. 2012, Harris et al. 2015). Two control rats that moved more than two thirds of a voxel (voxel size: 0.469 x 0.469 x 1mm) were removed from the analysis.

|  | **Control**  **(n = 14)** | **Control**  **‘movers removed’ (n = 12)** | **BDNF^+/-^**  **(n = 13)** |
| --- | --- | --- | --- |
| Mean translation (mm) | 0.32 ± 0.1 | 0.23 ± 0.05 | 0.21 ± 0.06 |
| Mean rotation (mrads) | 2.9 ± 0.4 | 2.4 ± 0.4 | 1.8 ± 0.4 |

**Table S2**

**Parameter estimates (± SEM) from the primary visual cortex and superior colliculus region for control and BDNF^+/-^ rats.**

|  | **Control**  **(n = 12)** | **BDNF^+/-^**  **(n = 13)** |
| --- | --- | --- |
| Primary visual cortex; parameter estimate | 1.9 ± 0.5 | 0.8 ± 0.5 |
| Superior colliculus;  parameter estimate | 1.9 ± 0.6 | 1.0 ± 0.7 |

**Table S3:** Behavioral parameters (± SEM) on the open arm during elevated plus maze testing (EPM) and plasma corticosterone (CORT) levels immediately following testing for control (n = 10) and BDNF^+/-^ rats (n = 11).

|  | **EPM**  **% distance (Open/total)** | **EPM**  **% time**  **(Open arms)** | **EPM**  **No. crossings (Open arms)** | **CORT**  **(nmol/L)** |
| --- | --- | --- | --- | --- |
| Control | 11.6 ± 3 | 13.1 ± 3 | 7.2 ± 1 | 208.0 ± 62 |
| BDNF^+/-^ | 14.2 ± 3 | 16.0 ± 3 | 7.5 ± 1 | 303.5 ± 83 |

**FIGURES**


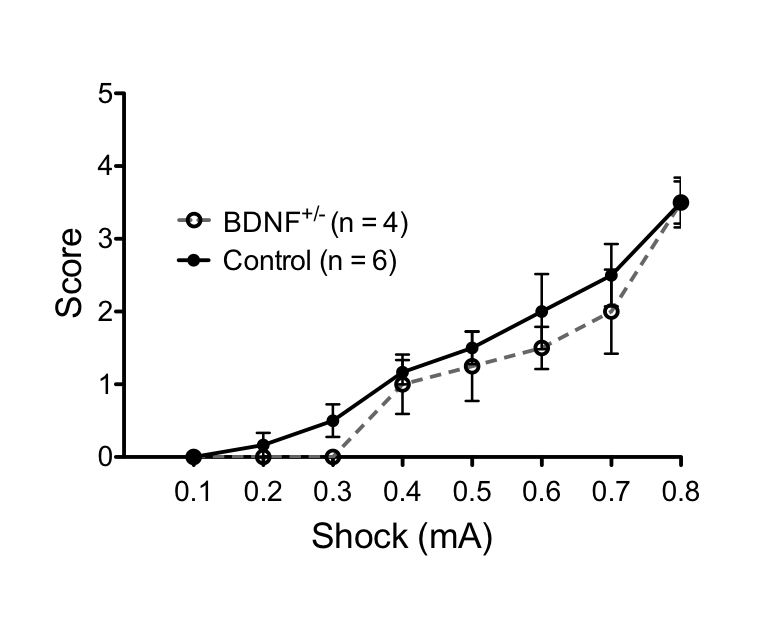


**Fig. S1: Foot-shock sensitivity in control and BDNF^+/-^ rats**

Behavioural score (± SEM) during shock titration. The response of the rat was scored as follows: 0 = no response; 1 = slight flinch; 2 = flinch/tail flick 3 = fast walking around chamber; 4 = jumping up with all feet off the grid floor; 5 = jumping up with vocalization and fast walking. 0.8mA was used in the main experiment.

**
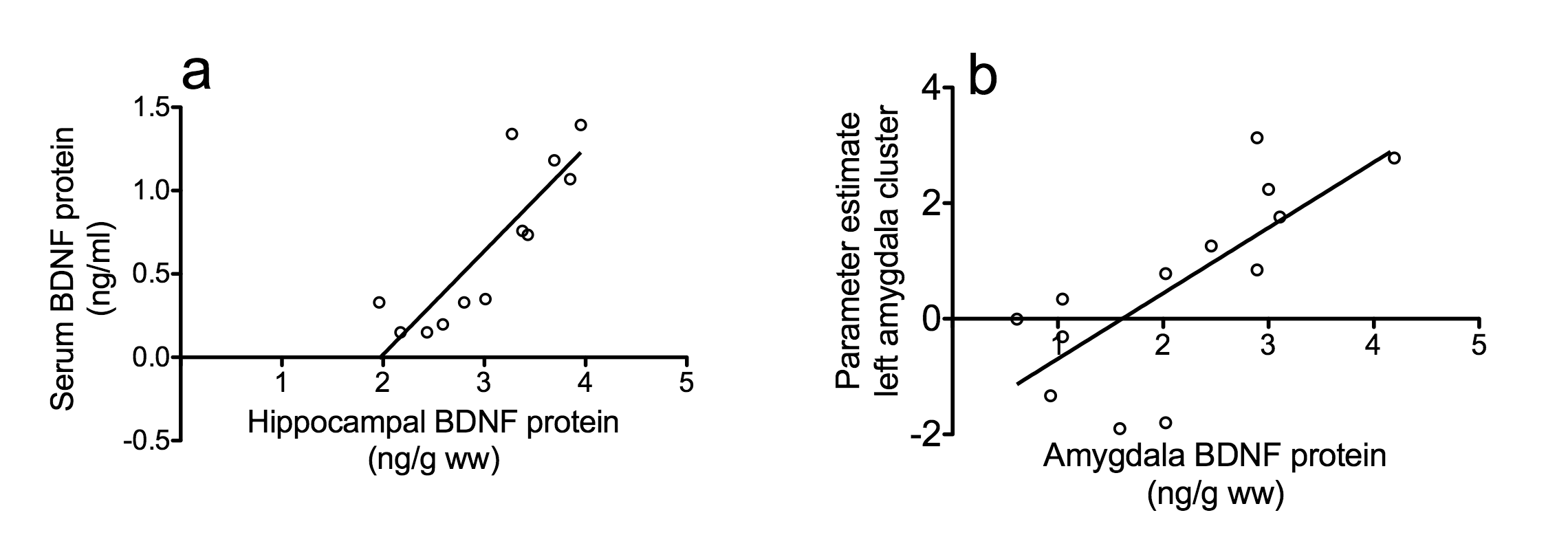
**

**Fig. S2: BDNF correlations**

a) There was a significant positive correlation between serum and hippocampus BDNF levels (Pearson’s correlation coefficient: r = 0.86, 95% CI [0.55, 0.96]; *P* = 0.0004) and b) there was a significant positive correlation between the extracted parameter estimates from the functional left amygdala cluster and baseline amygdala BDNF levels (measured 2 weeks after fMRI; Pearson’s correlation coefficient r = 0.73, 95% CI for slope [0.3, 0.9]; *P* = 0.0044).

**REFERENCES:**

Baran, S. E., Armstrong, C. E., Niren, D. C., Hanna, J. J. and Conrad, C. D. (2009) Chronic stress and sex differences on the recall of fear conditioning and extinction. *Neurobiol Learn Mem* **91**, 321-330.

Elfving, B., Plougmann, P. H. and Wegener, G. (2010) Detection of brain-derived neurotrophic factor (BDNF) in rat blood and brain preparations using ELISA: Pitfalls and solutions. *J Neurosci Methods* **187**, 73-77.

Ferris, C. F., Kulkarni, P., Sullivan, J. M., Harder, J. A., Messenger, T. L. and Febo, M. (2005) Pup suckling is more rewarding than cocaine: Evidence from functional magnetic resonance Imaging and three-dimensional computational analysis. *J Neurosci* **25**, 149-156.

Harris, A. P., Lennen, R. J., Marshall, I., Jansen, M. A., Pernet, C. R., Brydges, N. M., Duguid, I. C. and Holmes, M. C. (2015) Imaging learned fear circuitry in awake mice using fMRI. *Eur J Neurosci*, e-pub ahead of print 5 May; doi: 10.1111/ejn.12939.

Paxinos, G. and Watson, C. (2004) *The rat brain in stereotaxic coordinates - the new coronal set,* 5th Edition ed.*,* Academic Press.

Rorden, C. and Brett, M. (2000) Stereotaxic display of brain lesions. *Behav Neurol* **12**, 191-200.

Van Dijk, K. R. A., Sabuncu, M. R. and Buckner, R. L. (2012) The influence of head motion on intrinsic functional connectivity MRI. *NeuroImage* **59**, 431-438.
